# SARS-CoV-2 mRNA Vaccination in People with Multiple Sclerosis Treated with Fingolimod: Protective Humoral Immune Responses May Develop after the Preferred Third Shot

**DOI:** 10.3390/vaccines10020341

**Published:** 2022-02-21

**Authors:** Lutz Achtnichts, Arkady Ovchinnikov, Barbara Jakopp, Michael Oberle, Krassen Nedeltchev, Christoph Andreas Fux, Johann Sellner, Oliver Findling

**Affiliations:** 1 Department of Neurology, Aarau Cantonal Hospital, 5000 Aarau, Switzerland; lutz.achtnichts@ksa.ch (L.A.); arkady.ovchinnikov@ksa.ch (A.O.); krassen.nedeltchev@ksa.ch (K.N.); 2 Department of Infectious Diseases and Hospital Infection Prevention, Aarau Cantonal Hospital, 5000 Aarau, Switzerland; barbara.jakopp@ksa.ch (B.J.); christoph.fux@ksa.ch (C.A.F.); 3 Institute for Laboratory Medicine, Aarau Cantonal Hospital, 5000 Aarau, Switzerland; michael.oberle@ksa.ch; 4 Department of Neurology, University of Bern, 3012 Bern, Switzerland; 5 Department of Neurology, Landesklinikum Mistelbach-Gänserndorf, 2130 Mistelbach, Austria; johann.sellner@mistelbach.lknoe.at; 6 MS Center and Research Center for Clinical Neuroimmunology and Neuroscience Basel (RC2NB), Head, Spine and Neuromedicine, Clinical Research and Biomedicine and Biomedical Engineering, University Hospital and University of Basel, 4031 Basel, Switzerland

**Keywords:** SARS-CoV-2, S1PR-modulator, fingolimod, humoral immune response, vaccination, COVID-19

## Abstract

Evidence suggests limited development of protective IgG responses to mRNA-based vaccines in sphingosine-1-phosphate receptor (S1PR)-modulator treated individuals with multiple sclerosis (MS). We studied the extent of the humoral immune response after the preferred third mRNA SARS-CoV-2 vaccine in S1PR-modulator treated people with MS (pwMS) and insufficient IgG responses after the standard immunization scheme. Eight pwMS that were treated with fingolimod received a third homologous SARS-CoV-2 mRNA vaccine dose, either the Moderna’s mRNA-1273 or Pfizer-BioNTech’s BNT162b2 vaccine. We quantified the serum levels of IgG antibodies against the receptor-binding domain of SARS-CoV-2 four weeks later. An antibody titer of 100 AU/mL or more was considered protective. After the third vaccination, we found clinically relevant IgG titers in four out of eight individuals (50%). We conclude that the humoral immune response may reach protective levels after the third preferred dose of the homologous SARS-CoV-2 mRNA vaccine. Vaccine shots in S1PR-modulator treated pwMS ahead of schedule may be a strategy to overcome insufficient humoral immune responses following the standard vaccination scheme.

## 1. Introduction

Coronaviruses are a diverse group of viruses capable of infecting various animal species, and they can cause mild to severe respiratory infections in humans. Severe acute respiratory syndrome coronavirus 2 (SARS-CoV-2) is a highly transmissible and pathogenic coronavirus that emerged in late 2019 and has caused a pandemic of acute respiratory disease, termed “coronavirus infectious disease 2019” (COVID-19). This multi-organ disease poses an ongoing global threat to economics and public health [1]. Therefore, mass vaccination against SARS-CoV-2 is regarded as crucial to get a grip on the pandemic [2,3]. However, ongoing vaccine hesitancy, the highly transmissible nature with emerging mutations of the virus, and inconsistent public health measures represent hurdles to the success of this strategy [4,5].

The mRNA-based BNT162b2 (Pfizer–BioNTech) and mRNA-1273 (Moderna) vaccines elicit an immune response with nucleoside-modified mRNA encoding for the prefusion spike glycoprotein of SARS-CoV-2 and were shown to be effective in phase three trials [6,7]. The introduction in Switzerland started at the end of 2020, and both SARS-CoV-2 vaccines remained the only preparations available in Switzerland until autumn 2021. Early in the pandemic, it became evident that special considerations needed to be made for people with multiple sclerosis (pwMS), a chronic immune-mediated disease of the central nervous system (CNS) [8]. Older age and comorbidities, including obesity, progressive forms of MS, and a higher degree of disability, are risk factors for severe COVID-19 outcomes among pwMS. In addition, B-cell targeting treatments, such as CD20-depleting antibodies (e.g., rituximab, ocrelizumab) may increase the susceptibility to contracting COVID-19 and were repeatedly associated with higher rates for hospitalization, intensive care unit admission, and death [9,10,11,12,13]. In contrast, at an early pandemic stage, immunosuppression with ocrelizumab or teriflunomide has even been associated with milder COVID-19 courses [14,15]. The impact of treatments targeting the sphingosine 1-phosphate receptors (S1PR) expressed on lymphocytes is a matter of ongoing research as they reduce the egress of autoreactive T lymphocytes and their naïve progenitors from secondary lymphoid organs into the circulation. Notably, some immunotherapies approved for the treatment of MS attenuate the success of vaccinations [16]. Indeed, several reports corroborated that vaccination on treatment with S1PR modulators are associated with reduced seroconversion rates and attenuated responses [17,18,19]. This observation was also extended to mRNA vaccines against SARS-CoV-2 [20,21,22,23,24].

On the other hand, the risk of more severe COVID-19 in patients receiving S1PR modulators appear similar to that in the general population and the MS population with COVID-19 [25]. PwMS have a higher risk for getting bacterial and viral diseases, which can cause relapses of MS symptoms in the form of pseudo-relapses [26]. In August 2021, the Multiple Sclerosis Society of Switzerland recommended an additional preferred vaccine dose in pwMS receiving immunocompromising medication like S1PR modulators and an insufficient humoral immune response after the standard vaccine scheme with two doses as in these patients, the risk for infections is increased [27,28,29]. 

We report the rates of seroconversion in pwMS receiving S1PR modulators after the third dose of mRNA vaccine against SARS-CoV-2 in pwMS who failed to develop a sufficient IgG response after the standard vaccine scheme with two doses. This study is part of an ongoing project aiming at defining the risk of patients treated with immunomodulatory drugs for less effective vaccination. Previously, we reported data about the effect of mRNA vaccines against SARS-CoV-2 in pwMS treated with CD20-depleting agents [30]. 

## 2. Materials and Methods

At the MS outpatient clinic of the Cantonal Hospital Aarau, Switzerland, we performed a cross-sectional study of all pwMS receiving S1PR modulators. All patients gave written consent. The study was authorized by the ethical committee responsible for Northwest and Central Switzerland (permit number 2016-02233).

### 2.1. Data Collection

Between August and November 2021, we reviewed the patient records of all pwMS who were receiving S1PR modulators. We used descriptive statistics for data analysis. For group comparisons, we performed the Mann–Whitney U test. We operated version 9 of GraphPad Prism for Windows (www.graphpad.com accessed on 11 November 2021, GraphPad Software, La Jolla, CA, USA) for the statistical analysis.

### 2.2. Antibody Detection

We used Abbott’s SARS-CoV-2 IgG II Quant Assay (Abbott Ireland, Diagnostics Division, Ireland) to measure antibody titers. This chemiluminescent microparticle immunoassay was used for the quantitative and qualitative evaluation of IgG anti-spike antibodies binding to the receptor-binding domain (RBD) of the S1 subunit of the SARS-CoV-2 spike protein in human plasma and serum. The assay can detect past infections and quantify vaccine responses. The assay was performed following the manufacturer’s instructions. 

### 2.3. Clinical Procedure

At the suggestion of the Swiss Multiple Sclerosis Society, we offered all patients who received S1PR modulators to study the development of anti-spike IgG after two doses of vaccination against SARS-CoV-2 [27]. We drew blood at the earliest 28 days after the second shot to assess anti-spike-IgG development. For patients who had an insufficient IgG response, we offered a third shot. We re-evaluated the titer 28 days after the third shot. Against an official cut-off level of 50 AU/mL, we considered, according to suggestions, an IgG titer exceeding 100 AU/mL as sufficient [31,32]. According to the aforementioned official suggestions, the patients received the same vaccine as the first two doses for the third shot [33]. 

## 3. Results

We identified 29 pwMS treated with the S1PR-modulator fingolimod (FTY, Novartis, Basel, Switzerland) who were examined for seroconversion after the standard two shots of the BNT162b2 or mRNA-1273 vaccines. None of the patients suffered COVID-19 infection before or after vaccination. In 27 patients, complete datasets about vaccination and disease history were available. Eleven pwMS (41%) received the mRNA-1273 and 16 pwMS (59%) were vaccinated with BNT162b2. Since treatment initiation with FTY, the median time was 6.4 years (range 0.9 to 10.5). Table 1 displays the lymphocyte counts at the time point of antibody assessment and demographical data of all patients in the middle part.

We detected seroconversion after the standard vaccination scheme in all individuals. However, the antibody levels reached levels regarded as protective in only 6/27 (22%). Three of them received mRNA-1273, and three received BNT162b2. Group comparison between patients with sufficient antibody response and those with insufficient humoral response revealed no differences regarding age, disease duration, EDSS, the time between second vaccination and antibody testing, and lymphocyte count at the time point of antibody testing. All patients had the lymphopenia expected with FTY therapy.

Eight of 21 (38%) patients decided on a third shot, which was done with the same product as the initial two doses. Six patients (75%) received the BNT162b2 vaccine and two patients (25%) the mRNA-1273. Between the second and the third vaccination, the median time was 17 weeks (range 7.7 to 29.6). Table 1 displays the lymphocyte counts at the time point of antibody assessment and the demographical data of all patients in the right part. At the third shot, the median lymphocyte count was 0.6 G/l (range 0.3–0.9, normal value 0.8–4 G/l). 

**Figure 1:**
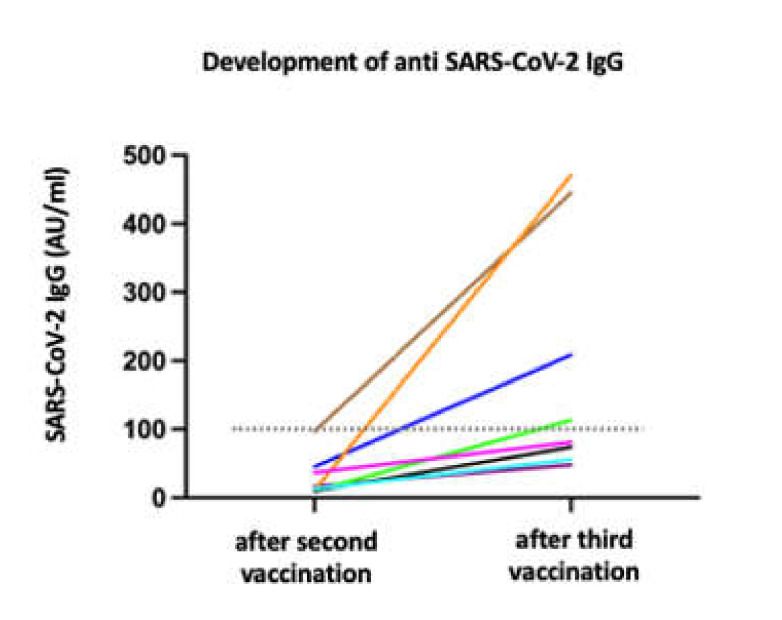
Increase of anti–receptor-binding domain (RBG) IgG antibody titers after the third vaccination against SARS-CoV-2. The horizontal dotted line indicates the cut-off for protection (100 arbitrary units (AU)/mL).

The analysis of the IgG response after the third vaccination shot ahead of schedule revealed that four patients (50%) had developed a protective humoral response (Figure 1). Two individuals received the mRNA-1273, and two individuals, the BNT162b2 vaccine. Comparison between patients with sufficient antibody response and those with insufficient humoral response revealed no differences in age, disease duration, EDSS, the time span between the second and the third shot, and lymphocyte count at the time point of third vaccination or antibody testing. 

## 4. Discussion

As part of an ongoing project to determine the risk of less effective vaccination in patients treated with immunomodulatory drugs in this monocentric evaluation, we studied the IgG response against the spike protein after a second and third shot of an mRNA-based vaccine against SARS-CoV-2 in pwMS receiving the S1PR-modulator FTY. We have previously reported on the effect of mRNA vaccines against SARS-CoV-2 in pwMS treated with CD20-depleting agents [30]. After the second dose, all pwMS receiving FTY had a positive humoral immune response (100%) but only 6 out of 27 patients (22%) reached levels considered as protective. We found no association between humoral response and age, disease duration, EDSS, the time span between the second shot and the antibody test, and the lymphocyte count at the time of the antibody test. The rate of seroconversion is comparable to previously reported low seroconversion rates from Israel (3.8%) and Italy (14%) but is higher than in patients treated with CD20-depleting agents [20,24,30]. Consequently, we followed the national recommendation and proceeded with a third vaccination ahead of schedule to increase the rate of individuals with humoral immune responses deemed protective [27,34]. We observed a further increase in IgG levels in all patients with previously insufficient humoral responses to the spike protein after two shots of mRNA vaccination. However, the threshold of 100 AU/mL for a protective humoral immune response was surpassed only in four out of eight individuals (50%). Thus, we fill the knowledge gap on the consequences of a third SARS-CoV-2 vaccination ahead of schedule in pwMS on fingolimod. Our findings are only partly comparable to recent data from a French study of individuals with kidney transplantation. A sufficient humoral response was achieved in that cohort with the third shot in 49% who did not develop adequate IgG antibodies after the standard vaccination scheme with mRNA-1273 [35]. The immunosuppressive maintenance therapy in these individuals included tacrolimus plus mycophenolate and steroids in 52.8%, and the cut-off for protective IgG was set at 50 AU/mL. However, compared to patients treated with CD20-depleting agents, there seems to be a larger proportion of patients receiving FTY with a sufficient humoral immune response [30]. However, due to the small number of patients, these data must be considered preliminary. 

Attenuated humoral immunity after vaccination is common in pwMS treated with S1PR modulators, and our findings are in line with previous results on vaccines other than against COVID-19 [17,19]. Individuals who do not develop sufficient humoral immune responses may still be protected by virus-specific T cell immunity [36]. Indeed, even in the absence of antibodies, robust and early T cell responses are present in mild or asymptomatic COVID-19 infection [37]. Moreover, there is evidence that T cells step up in pwMS on CD20 depletion if a humoral immune response to SARS-CoV-2 vaccination does not emerge [38,39,40]. Therefore, low or absent antibody titers cannot be equalized with lacking immunity against SARS-CoV-2. Although, due to the mode of action of S1P-modulators, which is based on sequestering autoreactive T lymphocytes in the lymphoid organs to reduce their recirculation and subsequent infiltration into the central nervous system, it must be suspected that the T cell response is impaired [41]. Thus, the recent report of very low rates of specific T cell responses after mRNA vaccination in pwMS on therapy with fingolimod (14.4%) in contrast to healthy controls (100%) deserves attention [24]. In that study, the neutralizing capacity of RBD-IgG of pwMS was moderate, in contrast to a strong correlation in healthy controls. Regardless, our data show that all patients who received the third vaccination had an increase in antibody titers, but this only reached a sufficient level of 50%. Weaknesses of our study are the low number of patients, the observational, non-randomized study design, and the lack of detection of nucleocapsid antibodies to rule out subclinical infection with SARS-CoV-2. However, a recently published study found nucleocapsid antibodies in only 6% of patients treated with rituximab. These data suggest that subclinical infection with SARS-CoV-2 is rare in immunocompromised patients [42]. The missing data on the T cell response is the most relevant weakness of our study. In turn, our data allows us to assume that even after a third vaccination, a relevant portion of patients may be less protected compared to healthy persons or pwMS treated with other therapies. Therefore, as there is an initial yet inconclusive indication of a possible relationship between antibody levels against the spike protein and protection from COVID-19 or a severe course, factors influencing the production of antibodies and sufficient cellular immune response should be evaluated [31]. 

In patients treated with anti-CD20 antibodies, stretching the interval between infusions with anti-CD20 antibodies and/or between the vaccine shots is discussed to increase the chances of successful anti-SARS-CoV-2 vaccination [43]. However, this approach is not possible with daily oral S1PR modulators. The possibility of interrupting or pausing therapy to achieve a better response to vaccination is certainly not a viable option, given the risk of significant disease rebound [44]. Therefore, studies on existing immunological protection against infection or protection against severe courses of COVID-19 after regular vaccination in patients treated with S1PR modulators, and whether other vaccination regimens are more successful, are urgently needed. Current data suggests that a heterologous vaccine regimen may be highly immunogenic, perhaps even more immunogenic than homologous regimens [45,46,47]. In addition, a recent study has shown that BNT162b2 in double doses elicits a stronger humoral response than the permutation of BNT162b2 and ChAd [47]. To answer these questions is relevant, given that S1PR modulators are a mainstay of multiple sclerosis treatment, and prevention of infections is of great importance for pwMS. Furthermore, current data show in healthy individuals that antibody titers decline in the months following the second vaccination, and it is unclear whether this decline is accelerated in immunosuppressed patients [48]. There are considerations to administer a fourth vaccine dose six months after the third vaccination in immunocompromised patients [49]. Based on our data, consideration should be given to shortening the interval between the third and fourth vaccination in specific patient groups. Furthermore, these patients should be treated preferentially with antibody therapies or, if available in the future, with antiviral medicines. In future studies, nucleocapsid antibodies should be measured to exclude asymptomatic SARS-CoV-2 infection, affecting the antibody titer against the spike protein.

## 5. Conclusions

The third SARS-CoV-2 mRNA vaccination in pwMS treated with S1P1-modulators may raise the rate of individuals with protective IgG responses. Additional vaccine shots in S1P1-modulator treated individuals may be a strategy to overcome insufficient humoral immune responses following the standard vaccination scheme.

## Figures and Tables

**Table 1:** Demographics, disease characteristics and lymphocyte count in people with MS receiving the second and third SARS-CoV-2 mRNA vaccination.

	2nd SARS-CoV-2 mRNA Vaccination	3rd SARS-CoV-2 mRNA Vaccination
Patients	*n* = 27 (f = 24 (89%))	*n* = 8 (f = 7 (88%))
Mean age (SD)	48.0 (11.7)	48.1 (12.0)
Mean disease duration (SD)	14.5 (10.5)	12.8 (5.0)
Median EDSS (range)	2.0 (0–6.5)	2.3 (1.0–3.0)
Median time between last vaccination and antibody testing (range)	15.6 weeks (2.0–27.6) *	4.4 weeks (3.9–5.9) **
Median lymphocyte count at time of antibody testing (range)	0.5 * (0.3–1.2)	0.5 ** (0.2–1.0)

Table legend: f = female; SD = standard deviation; RRMS = relapsing-remitting MS; * median time between second vaccination and spike protein IgG testing, ** median time between third vaccination and spike protein IgG testing.

## Data Availability

The data that supports the findings of this study are available on reasonable request from the corresponding author.
